# Sleep quality and associated factors among adult people living with HIV on follow-up at Dessie Town Governmental Health Facilities Antiretroviral Therapy Clinics, Northeast, Ethiopia, 2020, a multicenter cross-sectional study

**DOI:** 10.1186/s12888-023-04619-w

**Published:** 2023-03-02

**Authors:** Fisha Alebel GebreEyesus, Fatuma Seid Degu, Yeneabat Birhanu Yohanes, Abere Woretaw Azagew

**Affiliations:** 1 Department of Nursing, College of Medicine and Health Sciences, Wolkite University, PO Box 07, Wolkite, Ethiopia; 2 Department of Adult Health Nursing, Wollo University, Dessie, Ethiopia; 3 Department of Surgical Nursing, University of Gondar, Gondar, Ethiopia; 4 Department of Medical Nursing, University of Gondar, Gondar, Ethiopia

**Keywords:** HIV/AIDS, Sleep disturbance, Quality of sleep, Dessie, Ethiopia

## Abstract

**Background:**

Poor sleep quality is an important health problem in people living with HIV. The exact cause of sleep disturbance is not well known, but it may relate to HIV itself, antiretroviral drug side effects, and other HIV-related disorders. As a result, the purpose of this study was to assess sleep quality and associated factors among adult HIV patients on follow-up at Dessie Town governmental health facilities’ antiretroviral therapy clinics in Northeast Ethiopia in 2020.

**Methods:**

A multi-center cross-sectional study was conducted among 419 adult people living with HIV/AIDS from February 1/2020 to April 22/2020 in Dessie Town governmental antiretroviral therapy clinics. A systematic random sampling method was used to select the study participants. An interviewer-administered method of data collection with a chart review was used. The Pittsburgh Sleep Quality Index was used to evaluate sleep disruption. A binary logistic regression was conducted to see the relationship between a dependent variable and independent variables. Variables with a *p*-value of < 0.05 and a 95% confidence interval were used to declare an association between factors and a dependent variable.

**Results:**

A total of 419 study participants were enrolled in this study, with a response rate of 100%. The mean age of the study participants was 36 ± 6.5 SD years and 63.7% of the participants were female. The prevalence of poor sleep quality was found to be 36% (95% CI, 31–41%). Being female (AOR = 3.45, 95% CI: 1.52–7.79), viral loads 1000 copies/ml (AOR = 6.88, 95% CI: 2.79–16.9), CD4 cell count 200 cells/mm3 (AOR = 6.85, 95% CI: 2.42–19.39), WHO stage II and III (AOR = 4.29, 95% CI: 1.05–17.53), having anxiety (AOR = 10, 95% CI: 4.21–23.9.

**Conclusion:**

The findings of this study showed that more than one-third of the study participants had poor-quality sleep at the Dessie Town Health Facility ART clinic. Being female, low CD4 cell counts, viral load ≥1000 copies/ml, WHO stage II and III, depression, anxiety, sleeping in a communal bedroom, and living alone were predictors of poor sleep quality.

**Supplementary Information:**

The online version contains supplementary material available at 10.1186/s12888-023-04619-w.

## Background

HIV/AIDS is one of the most overwhelming and devastating pandemics humanity has ever seen in recent history [1]. Globally, 38.4 million people were living with HIV in 2021 [2, 3]. Of this, HIV infection among adults accounts for 95.6% of people living with HIV (PLWHIV) and 85.3% of total HIV/AIDS deaths [3].

Ethiopia was one of the countries hardest hit by the HIV epidemic, which was characterized as mixed, with wide regional variations and concentrations in urban areas, including some distinct hotspot areas driven by key and priority populations [4, 5]. Sexually active adults (15–49 years) were the primary drivers of the HIV/AIDS epidemic and the primary target of national HIV/AIDS prevention and control efforts [6].

Sleep disturbance is a common complaint in individuals with chronic diseases, including HIV infection [7]. It occurs at all stages of infection but is more common in the advanced stage [8]. Previously published literature showed that PLWHIV experiences insomnia and other sleep difficulties at a greater rate than the general population, which ranges from 40 to 100% compared with the normal population of 13–30% [9–11].

A meta-analysis report found that the global pool prevalence of sleep disturbances among adult PLWHIV was 58% [12]. In Africa, there is a high prevalence of sleep disturbance among adult PLWHIV, which ranges from 39.4 to 94% [13–16]. In Ethiopia, nearly 50% of PLWHA developed mental health issues such as depression and anxiety, which further hampered sleeping quality [17]. However, awareness of sleep disturbance as a health issue in general and as an HIV-related health issue specifically is low among patients, as well as physicians, who may not always recognize the seriousness of disrupted sleep in a patient’s overall quality of life [7, 18].

Poor quality of sleep is a symptom characterized by difficulty in initiating and maintaining sleep, excessive somnolence, a disturbed sleep-wake schedule, and dysfunction associated with the sleep and sleep stages [19]. The causes of poor sleep quality in PLWHIV are not well understood, but previous research has suggested that monogenic cytokines such as tumor necrosis factor-alpha (TNF-alpha) and interleukin-1 (IL-1) are involved [20]. Other factors such as HIV, lower immunity, antiretroviral medication side effects [21–23], low CD4 cell count, efavirenz-based ART regimen, duration of living with HIV, stress, anxiety, and depression were all linked to sleep disturbances [24–28].

Sleep disruption leads to non-adherence to recommended medications [29] that accelerate disease progression to a fatal stage [30], decreased job performance, absence from work, being more prone to accidents, decreased quality of life, increased health care costs, a high rate of psychiatric co-morbidities [31], altered cognitive functioning [32], increasing the risk of developing hypertension [33], being overweight [34], and increasing unprotected sex. Whereas better sleep quality in HIV-infected individuals is associated with indicators of quality of life such as general well-being, less anxiety, fewer depressive symptoms, and lower symptom severity [35, 36],

The simultaneous occurrence of poor quality of sleep and HIV infection makes clinical management more complicated, so understanding the magnitude and major factors of poor quality of sleep is important to identify and treat mental health problems as early as possible. Although the magnitude of poor sleeping quality is reportedly high among HIV-infected persons, relatively few studies have been undertaken nationally. Therefore, this study aimed to assess sleep quality and associated factors among adult people living with HIV on follow-up at Dessie town governmental health facilities (Antiretroviral Therapy Clinics, Northeast, Ethiopia, 2020).

## Methods

### Study setting

A multi-center cross-sectional study was conducted among adult PLWHIV on follow-up at Dessie Town Governmental Health Facilities Antiretroviral Therapy Clinics from February 1, 2020, to April 22, 2020. Dessie town is found in the Amhara regional state, which is 451 km from Addis Ababa. In Dessie Town, there are three private hospitals, six government health centers, and one government hospital. Dessie Referral Hospital, Banbuawuha Health Center, Segno Gebaye Health Center, and Dessie Health Center were the government health facilities that gave ART services to communities. Those health facilities serve different zones; such as the Oromia special zone, South Wollo, North Wollo, and partial parts of North Showa. Currently, there are approximately 9590 adult HIV-positive patients enrolled in the ART clinic. All adult PLWHIV who were attending each ART clinic were considered the source population, whereas those adult PLWHIV who were attending the ART clinics during the data collection period were taken as the study population.

### Sample size and sampling procedure

**Fig. 1:**
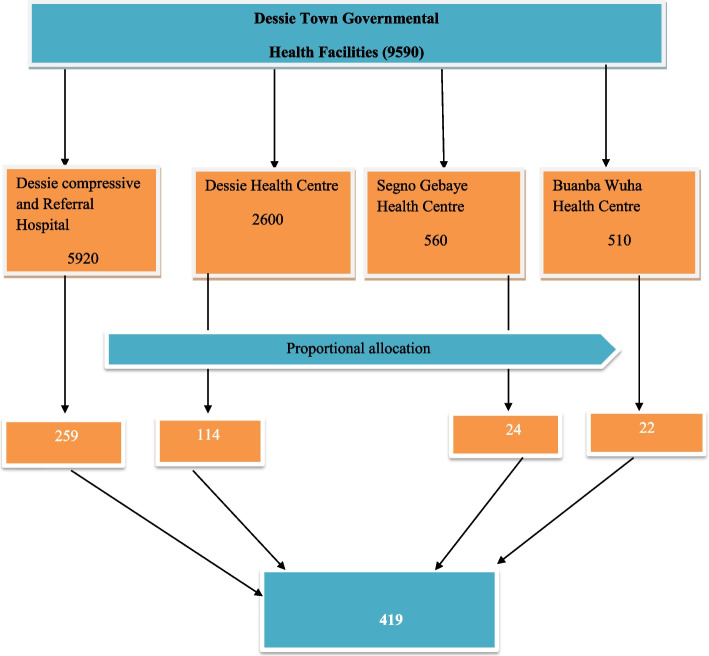
Proportional allocation of sample size among PLWHIV on follow-up at Dessie Town governmental health facilities, ART clinic Northeast, 2020 (*n* = 419)

The sample size was determined using a single population proportion formula considering the assumptions of a 95% CI, a 45.8% population proportion from the previous study [33], and a 5% margin of error. Taking a 10% nonresponse rate, the final sample size was 419. A systematic random sampling technique was used to select study participants from adult HIV-positive clients who visited the ART clinic. Initially, the sampling interval was determined by dividing the total number of adult HIV-positive clients who visited the ART clinic during the data collection period by the calculated sample size for each health facility. After determining the k interval, the first participants were selected by using the lottery method, and the subsequent sample was taken based on the k interval until the required sample was reached. The calculated sample size was proportionally allocated to each selected health facility. Dessie Comprehensive and Referral Hospital has 259 beds, Dessie Health Center has 114 beds, Segno Gebaye Health Center has 24 beds, and Buanba Wuha Health Center has 22 beds (Fig. 1).

### Data collection tools and procedures

A structured interviewer-administered questionnaire with a chart review was employed to collect data. The questionnaire was first developed in English and translated to Amharic (local language) during data collection and back to English for analysis to maintain its consistency. The questionnaire includes questions on sociodemographic factors, clinical factors, personal and behavioral factors, psychological factors (stress, anxiety, and depression), and poor-quality sleep-related questions. A validated Pittsburgh Sleep Quality Index (PSQI) tool was used. Its Cronbach’s alpha is 0.88 [37, 38]. Sleep disturbance was assessed with a 19-item questionnaire with 7 components such as subjective sleep quality, sleep latency, sleep duration, habitual sleep efficiency, sleep disturbances, use of sleeping medications, and daytime dysfunction during the last month. Participants with a PSQI global score of > 5 were considered to have poor sleep quality, whereas those with a PSQI global score of 5 were considered to have no poor sleep quality [39].

The perceived stress scale (PSS) was used to assess the client’s stress condition. It had 10-item stress scales ranging from 0 to 4 points on the Likert scale, with a score of a minimum of 0 and a maximum of 40. The participants had a mean score of 0–13, which was classified as low stress; 14–26 as moderate stress, and 27–40 as severe stress [40].

Anxiety was measured using the hospital anxiety scale. The tool had seven Likert scales ranging from 0 to 3, with a minimum of 0 and a maximum of 21 scores, and the higher score indicated having anxiety [41]. Depression was measured using the hospital depression scale. It used a 7-point Likert scale, with a minimum score of 0 and a maximum score of 21; the lower score indicates no depression, and the higher score indicates depression. Participants with a mean score of 8 or higher were considered to be depressed, while those with a score of 8 or lower were considered to be depressed. Participants with a mean score of 8 or higher were considered to have anxiety [8, 41]. Furthermore, the clinical characteristics such as CD4 count, duration of HIV diagnosis, ART regimen, viral load, duration of ART use, WHO treatment stage (T-stage), comorbidity, and opportunistic infection of the study participants were retrieved from patient records using a standardized checklist. Four BSc nurses as the data collectors and one MSc supervisor were used. The data were collected from February 1/2020 to April 22/2020.

### Data processing and analysis

The data were coded and entered into Epi Info Version 7 before being exported to SPSS Version 20 for analysis. Descriptive and analytical statistical procedures were used. Descriptive statistics such as percentage, mean, median, standard deviation, and interquartile range (IQR) were used. Tables and bar graphs were also used for data presentation. A binary logistic regression model was used to identify factors associated with poor sleep quality. Those variables that showed a statistically significant value at a *p*-value of < 0.25 in the bivariable analysis were entered into the multivariable logistic regression model to control the possible effect of confounders. Variables with a *p*-value of ≤0.05 with a 95% confidence interval in multivariable logistic regression were considered statistically significant. Model fitness was checked using the Hosmer and Lemeshow goodness of fit test.

## Results

### Socio-demographic characteristics of the study participants

A total of 419 participants were enrolled in the study, giving it a 100% response rate. The mean age of the participants was 36 ± 6.5 SD years. Nearly two-thirds (63.7%) were females. Among study participants, 180 (43%) were married, 242 (57.8%) were Muslim, 149 (35.6%) were attending primary school, 357 (85.2%) were urban dwellers, and 108 (25.8%) were currently in daily labor. The mean monthly family income was 800 Ethiopian Birr (Table 1).

**Table 1:** Socio-demographic related characteristic of PLWHIV on follow-up at Dessie governmental health facilities ART clinics, Northeast Ethiopia, 2020 (*n* = 419)

Variable	Category	Frequency(n)	Percent (%)
**Sex**	Male	152	36.3
	Female	267	63.7
**Age group**	18–35 years	207	49.4
	> 35 years	212	50.6
**Marital status**	Single	109	26
	Married	180	43
	Divorce	79	18.9
	Widowed	51	12.2
**Religion**	Orthodox	172	41.1
	Muslim	242	57.8
	Protestant	4	1.00
	Other	1	0.2
**Education**	No formal education	114	27.2
	Primary school	149	35.6
	Secondary school	95	22.7
	College and above	61	14.6
**Residence**	Urban	357	85.2
	Rural	62	14.5
**Occupation**	Student	64	15.3
	Daily labor	108	25.8
	Farmer	35	8.4
	Housewife	85	20.3
	Civil servant	55	13.1
	No job	16	3.8
**Monthly income**	≤ 1000 birr	266	63.5
	> 1000 birr	153	36.5

Other at religion = Catholic

### Clinical, behavioral, and mental health characteristics of PLHIV

Among study participants, 266 (63.5%) had been HIV-positive for more than 5 years, 114 (27.2%) were overweight, 393 (93.8%) were T-stage I, 258 (61.6%) had a CD4 count greater than 350 cells/mm3, and 255 (60.85%) had viral loads greater than 1000 copies/mL. Regarding ART status, 281 (67.1%) patients used the efavirenz-based regimen, and 266 (63.5%) had good ART adherence. Nearly 15 % of the study participants had comorbidity, and 6 % of the participants had a history of opportunistic infections. Regarding the mental health characteristics of the study participants, 128 (30.5%) had anxiety, 156 (37.2%) had depression, and 49 (11.7%) had severe stress. Fifty-five (13.1%) of the study participants chewed khat once in their lifetime, 46 (11%) were currently chewing khat, 30 (7.2%) were currently drunk; and 16 (3.8%) were currently smoking cigarettes (Table 2).

**Table 2:** The clinical, behavioral, and mental health characteristics of PLHIV on follow-up at Dessie town governmental health facilities ART clinics, Northeast Ethiopia, 2020 (*n* = 419)

Variable	Category	Number (n)	Percent (%)
**Duration HIV infection**	> 5 years	266	63.5
	≤ 5 years	153	36.5
**BMI**	18.5-25 kg/m^2^	255	60.9
	< 18 kg/m^2^	50	11.9
	> 25 kg/m^2^	114	27.2
**Current WHO staging**	Stage I	393	93.8
	Stage II	21	5.0
	Stage III	5	1.2
**Recent CD4 cell count**	350 cell/mm^3^	255	60.85
	200-350cell/mm^3^	80	19.09
	< 200cell/mm^3^	81	19.33
	Other	3	0.71
**Recent viral load**	<1000copy/ml	313	75.4
	≥1000 copies/ml	102	24.6
	Other	4	01
**Current ART regimen**	Efavirenz-based ART regimen	281	67.1
	Nevirapine-based ART regimen	100	23.9
	Lobenavier /Atazenavier based ART	38	9.1
**ART adherence**	Good	279	66.6
	Fair	70	16.7
	Poor	70	16.7
**History of comorbidity**	Yes	61	14.6
	No	358	85.4
**Current opportunistic infection**	Yes	26	6.2
	No	393	93.8
**Anxiety**	Yes	128	30.5
	No	291	69.5
**Depression**	Yes	156	37.2
	No	263	62.8
**Stress**	Low	73	8.8
	Moderate	333	79.5
	Sever	49	11.7
**Khat chewed status**	Never chewed	318	75.9
	Former chewed	55	13.1
	Current chewed	46	11
**Alcohol drink status**	Yes	30	7.2
	No	389	92.8
**Smoking status**	Never Smoked	381	90.9
	Former smoked	22	5.3
	Current smoked	16	3.8

### Sleep characteristics of the study participants (Table 3)

#### The prevalence of poor-quality sleep

**Fig. 2:**
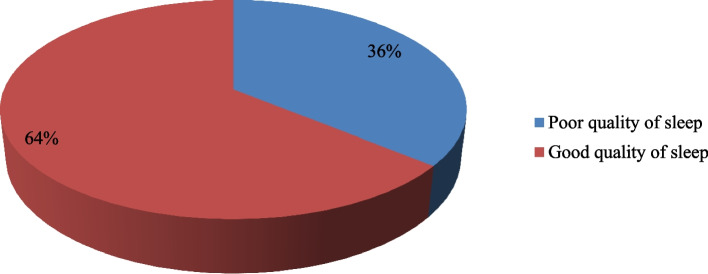
The prevalence of poor sleep quality among PLWHIV at Dessie Town governmental health facilities and ART clinics in the Northeast in 2020 (*n* = 419)

The prevalence of poor sleep quality among PLWHIV on follow-up was found to be 36% (95% CI; 31–41%), with males having 20% and females having slightly more than 45%. The median PSQI score was 3 (IQR: 0–17) (Fig. 2).

**Table 3:** Characteristics of sleep quality among PLWHIV on follow-up at Dessie town governmental health facilities, ART clinics, 2020 (*n* = 419)

Variable	Category	Frequency (n)	Percent (%)
**Subjective sleep** **Quality**	Very good	221	52.7
	Fairly good	80	19.1
	Fairly bad	40	9.5
	Very bad	78	18.6
**Sleep latency**	<15mints + not during the past month	170	40.6
	16–30 minutes once or twice a week	78	18.6
	31-60 min once or twice a week	51	12.2
	> 60 minutes 3 times a week	120	28.6
**Sleep duration**	> 7 hrs	251	59.9
	6–7 hrs.	47	11.2
	5–6 hrs	35	8.4
	< 5 hrs	86	20.5
**Habitual sleep** **Efficacy**	≥85%	319	76.1
	75–84%	17	4.1
	65–74%	30	7.2
	< 65%	53	12.6
**Sleep disturbance**	None	193	46.1
	1–9	207	49.4
	10–18	3	0.7
	19–27	16	3.8
**Used sleep medication**	Not during the last month	390	93.1
	less than once a week	12	2.9
	once or twice a week	6	1.4
	≥3 times a week	11	2.6
**Daytime dysfunction**	No problem	280	66.8
	Slight problem(1–2/week	109	26
	Moderate problem> 2/week)	21	5
	Big problem > 3/week	9	2.1
**Global sleep quality**		Median	IQR
		3	0–9

#### Characteristics of the sleep patterns of the study participants

The participants went to bed on average at 10:02 pm and awakened in the morning at 4: 08 am. The mean time sleeping each night was 7:02 hrs. (SD ±1.07 hours). Two hundred and twenty-one (52.7%) had very good sleep quality, 120 (28.6%) had slept for> 60 minutes, and 86 (20.5%) slept < 5:00 per 24:00 hours.

#### Reasons for difficulty in maintaining sleep

**Fig. 3:**
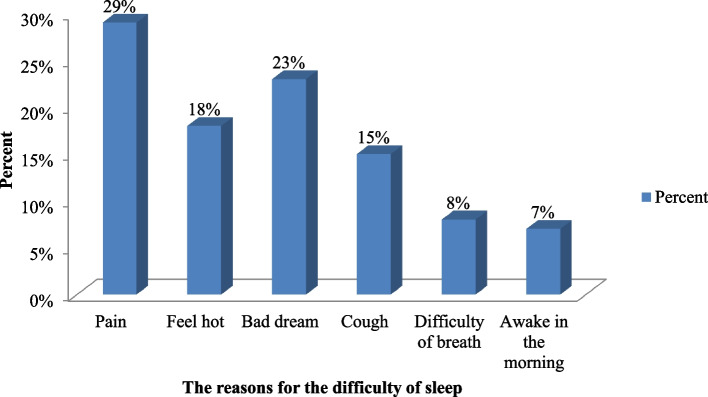
Sleep difficulties among PLWHIV on follow-up at Dessie Town governmental health facilities, ART clinics in the Northeast, 2020 (*n* = 419)

Above half (56.6%) of adult PLWHIV were facing difficulty maintaining sleep due to being unable to fall asleep within 30 minutes (Fig. 3).

#### Factors associated with poor sleep quality

To assess the association of different independent variables with quality of sleep, bivariable logistic regression analysis was conducted and for a crude association, all variables with a *p*-value less than 0.25 (*P*-Value< 0.25) were become a candidate for multivariable logistic regression. In multivariable analysis; being female (AOR = 3.45, 95% CI; 1.52–7.79), viral loads ≥1000 copies (AOR = 6.88, 95% CI; 2.79–16.9), CD4 cell < 200 cells/mm^3^ (AOR = 6.85, 95% CI; 2.42–19.39), WHO T-stage II and III (AOR = 4.29, 95% CI; 1.05–17.53), having anxiety (AOR = 10, 95% CI; 4.21–23.9), having depression (AOR = 4.44, 95% CI; 1.95–10.10), having separate bedroom (AOR = 3.94, 95% CI; 1.86–8.36), and living alone (AOR = 6, 95% CI; 2.81–13.12) were determinant factors of poor sleep quality in PLWHIV (Table 4).

**Table 4:** Factors associated with poor quality of sleep among PLWHIV on follow-up at Dessie town governmental health facilities, ART clinic 2020(*n* = 419)

	Poor quality of sleep		COR (95%CI)	AOR (95%,CI)
	Yes	No		
**Sex**				
Female	121	146	3.37 (2.114,5.373)	3.45 (1.52–7.79)*
Male	30	122	1	1
**Age**				
> 35 years	151	56	2.19 (1.45–3.3)	_______
≤ 35 years	117	95	1	
**Marital status**				
Married	64	116	1	
Unmarried	37	72	0.93 (0.56–1.53)	_________
Divorce	33	46	1.3 (0.75–2.23)	_________
Widow	17	34	0.47 (0.47–1.74)	_________
**Education**				
Secondary school	23	72	0.4 (0.2,0.8)	________
Primary school	46	103	0.56 (0.3–1.)	________
Unable to read and write	55	59	1 (0.6–2)	________
College and above	27	34	1	
**Residence**				
Rural	15	47	1	
Urban	136	221	1.9 (1.04–3.6)	_________
**Employment**				
Not active employment	21	59	1	
Active employment	130	209	1.7 (1–3)	_________
**Family average monthly income**				
> 1000 birr	63	90	1	
≤ 1000 birr	88	178	1.41 (0.938–2.136)	________
**BMI**				
18.5–25	67	188	1	
< 18.5	12	38	0.8 (0.4–1.8)	_________
> 25	72	42	4.8 (3–7.7)	_________
**Taking coffee and tea**				
No	69	137	1	
Yes	82	131	1.24 (0.83–1.85)	_________
**Smoking status**				
Never smoked	126	255	1	
Current smoked	11	5	4.4 (1.5–13)	_________
Former smoked	14	8	3.5 (1.4–8.7)	_________
Past smoked	14	8	3.5 (1.4–8.7)	_________
**Khat chewing**				
Never chewed	95	223	1	
Past chewed	27	28	2.26 (1.27–4)	________
**Drinking alcohol**				
No	136	253	1	
Yes	15	15	1.86(.88–3.92)	_________
**Current ART regimen**				
Nevirapine contain regimen	27	73	1	
Efaverized contain regimen	109	172	1.7 (1–2.8)	_________
Lobenavier/Atazenavier	15	23	1.8 (0.8–3.9)	_________
Contain regimen				
**ART drug adherence**				
Good	106	243	1	
Poor	45	25	4 (2.4–7)	_________
**Duration of HIV infection**				
≤ 5	44	109	1	
> 5 years	107	159	1.67 (1.1–2.6)	_________
**Viral loads**				
< 1000 copies/ml	77	236	1	1
≥ 1000 copies/ml	74	28	8.1 (4.88–13.42)	6.88 (2.79–16.9)**
**CD4 cells**				
> 350 cells/m3	66	192	1	1
200–350 cells/mm3	29	51	1.7 (1–2.8)	
< 200 cells/mm3	56	25	6.5 (3.8–11)	6.85 (2.422–19.39)**
**WHO stage**				
Stage I	138	255	1	1
Stage II&III	13	13	1.84(.833–4.09)	4.29 (1.05–17.53)^*^
**Opportunistic infection**				
No	137	256	1	
Yes	14	12	2.18 (0.98–4.84)	_________
**Depression**				
No	32	231	1	1
Yes	119	37	23 (13.8–39)	4.44 (1.95–10.1)^**^
**Stress**				
Low stress	10	27	1	
Moderate stress	107	226	1.28 (0.6–2.7)	_________
Severe stress	34	15	6.12 (2.38–15.8)	_________
**Anxiety**				
No	42	249	1	1
Yes	109	19	34 (18.9–61)	10 (4.212–23.93)^**^
**Discloser status**				
Yes	125	252	1	
No	26	16	3.3 (1.7–6)	__________
**Separate bedroom**				
Yes	35	185	1	1
No	116	83	7.4 (4.7–11.7)	3.94 (1.86–8.36)^**^
**Live alone**				
No	38	224	1	1
Yes	113	44	15.14 (9–24.7)	6 (2.81–13.12)^**^
**Comorbidity disease**				
No	118	240	1	
Yes	33	28	2.39 (1.4–4)	_________
**Sleep in a noisy environment**				
No	56	217	1	
Yes	95	52	7 (4.6–11)	_________

## Discussion

The findings of this study revealed that the prevalence of poor quality sleep among adult PLWHIV on follow-up was found to be 36% (95% CI: 31–41%). The prevalence of poor sleep quality among males was found to be 20%, whereas, among females, it is somewhat higher at 45%. The finding of this study was in line with a study conducted in the USA (40.93%) [26] and China (32.1% [27] where the prevalence of poor sleep quality was predominant in females**.** The finding of the study was lower than the study conducted in Ethiopia, Zewditu Memorial Hospital (56%) [17], Mettu Karl Referral Hospital (57.1%), Hawassa University Comprehensive Specialized Hospital (57.6%), Nigeria 45.8% [33], Cameroun 66.7% [44], China 43.1% [28], Iran 47.5% [45], Mexico (58.6%) [46], Germany 63% [47], and Paris 68% [39], 63% [48]. The possible discrepancy is due to variations in sociocultural characteristics, sampling methods, study setting, and design, type of measurement tools, and data collection methods. On the contrary, the finding of this study was higher than the study conducted in South Africa by 16% [49]. The possible reason for this discrepancy may be due to the former study’s use of longitudinal follow-up, which may have led to a loss of data.

The current study discovered that sex was a factor in poor sleep quality. When compared to their counterparts, women were 3.45 times more likely to have poor sleep quality (AOR = 3.45, 95% CI: 1.52–7.79). This study was supported by a study carried out in Ethiopia at Zewditu Memorial Hospital, which stated that poor sleep quality was significantly associated with female gender [AOR = 3.40, 95% CI: (1.80, 6.41)] among people with HIV/AIDS. Females may be more vulnerable to stress due to the high burden of household responsibilities and changes in hormonal levels, according to one possible explanation. There was a hormonal imbalance of estrogen and progesterone during the premenopausal and menopausal periods that decreased the level of estrogen as well as progesterone levels, resulting in a two-fold increase in the number of arousals after sleep and decreased total sleep time [17, 50].

Participants who had a viral load greater or equal to 1000 copies/mL were nearly 7 times more likely to develop poor sleep quality compared to those clients having viral loads less than 1000 copies/ml (AOR = 6.88, 95% CI; 2.79–16.9). This is supported by a study conducted in California [29]. The possible explanation could be due to high viral loads in the peripheral circulation enhancing HIV entry into the central nervous system, which activates macrophages and astrocytes [51] and consequently impairs their function, which decreases the release of sleep regulatory substances (TNF-alpha) [52]. Viral load increments are associated with the disease’s progression to the chronic stage, which changes sleep by increasing arousal and waking during sleep periods [53].

The odds of experiencing poor sleep quality among adult PLWHIV who were in WHO T-stage II were 4.29 times higher compared to those who were in WHO T-stage I (AOR = 4.29, 95% CI; 1.05–17.53). This is supported by a study conducted in UAS [54]. Patients with T-stage II and above may experience opportunistic infections that impair sleep quality.

Participants who had CD4 cell counts less than 200 cells/mm^3^ were nearly 7 times more likely to develop poor sleep quality compared to those having CD4 cell counts greater than 350 cells/mm^3^ (AOR = 6.85, 95% CI; 2.42–19.39). This is supported by a study conducted in Ethiopia [17], Nigeria [24], Mexico [46], and the USA [55]. Immune declines associated with HIV infections are directly linked to the psyche by a complex network of nerves, hormones, and neuropeptides. This network has a direct impact on sleep [33]. With the progression of HIV, it is known that viral load increases and CD4+ cell count decrease; as a result, the quality of sleep worsens along the course of the disease, and this is related to CD4+ cell count and viral load as well [56].

Participants who had depression were 4.44 times more likely to develop poor sleep quality compared to those who had no depression (AOR = 4.44, 95% 1.95–10.10). This is supported by a study conducted in Ethiopia [17, 43], China [28], Germany [47], and five cities in the USA [26]. For example, a study carried out at Zewditu Memorial and Hawassa Hospital showed that depressed individuals were 4 times and 5 times more likely to experience poor sleep quality compared to their counterparts, respectively [17, 43]. The possible explanation could be due to links between sleep, emotional regulation, changes in the hypothalamic-pituitary-adrenal axis, the involvement of psychopathology and the sleep-wake cycle, and decreased serotonin neurotransmitters, which result in impaired cognitive performance and disrupt normal sleep patterns. Moreover, studies conducted in the USA and China [28, 57] showed that the strongest factor associated with poor quality of sleep was depression, and that psychological morbidity is a major factor in sleep disturbances among HIV-infected patients. Given the likely bidirectional association between sleep and depression, targeted management of one may improve the other [28]. Therefore, treating depression might improve sleep quality, and addressing sleep disturbances may relieve psychological morbidity.

In the current study, participants who had anxiety were 10 times more likely to develop poor sleep quality compared to those participants with no anxiety (AOR = 10, 95% CI; 4.21–23.9). This is supported by a study conducted in Ethiopia [43], China [28], and the USA [57]. The reason is that, according to the polysomnographic features that characterize patients with anxiety have longer sleep onset latency, a greater number of arousals, and greater wake time during the night, with fewer transitions into non-REM sleep [58].

In the present study, participants who lived alone were 6 times more likely to develop poor sleep quality compared to those living with their families (AOR = 6, 95% CI; 2.81–13.12). This is supported by a study conducted in Ethiopia [42], and the USA [59]. Physical and social aspects of sleeping arrangements have negatively affected sleep quality [56]. Better family and social support were associated with better sleep quality. Living with a supportive family can have a positive effect on mood, prevent social isolation, and promote healthy sleep habits. Moreover, social support may help maintain a more consistent and consolidated sleep-wake schedule and may affect sleep by attenuating the effects of psychological stress on sleep [25, 60].

In this study, participants who did not sleep in separate bedrooms were nearly four times more likely than those who did (AOR = 3.94, 95% CI: 1.86–8.36). This is supported by a study conducted in the USA [59]. Sleep can be disrupted by a variety of factors related to the location of the bedroom in the house. The absence of separate sleeping rooms predisposed extra sound and light, a sense of insecurity, an image or art, and a lack of privacy to be negative influences on sleep quality [61];

### Limitation

Variables such as sleep with partners and family size may affect sleep quality, but this issue did not incorporate in the current study, and Substances (Alcohol, cigarette, and khat) use was not measured quantitatively. We had constructing just one multivariable model and entering any variables that were significant in univariable and bivariable analysis which may lead to bias for estimates of association. Moreover, our study is cross-sectional, which is weak to evaluate the cause-effect relationship and small sample size (which may have led to the wide confidence intervals for all associations).

## Conclusion

The findings of this study showed that more than one-third of the study participants had poor-quality sleep at the Dessie Town Health Facility ART clinic. So, healthcare providers working in the ART care service should give strong attention to female patients, people living with HIV/AIDS who have a low CD4 cell count < 200 cells/mm3) and a viral load ≥1000 copies/mL) in assessing sleep quality, Aside from that, we should care for people suffering from mental illnesses such as depression and anxiety, offer counseling services to clients, have a separate bedroom, and emphasize the importance of social support, especially when living with their families.

## Supplementary Information

**Additional file 1.** English version Questionnaire.

## Data Availability

The data will be made on request via the corresponding author’s email.

## Abbreviations

ART: Antiretroviral Therapy
BMI: Body Mass Index
E.C: Ethiopian Calendar
HADS: Hospital Anxiety- Depression Scale
NSP: National Strategic Plan
PLWHIV: People Living with HIV
PSQI: Pittsburgh Sleep Quality Index
UNAIDS: United Nations Programme on HIV/AIDS
WHO: World Health Organization
