# Supplementary material for: Sleep quality and associated factors among adult people living with HIV on follow-up at Dessie Town Governmental Health Facilities Antiretroviral Therapy Clinics, Northeast, Ethiopia, 2020, a multicenter cross-sectional study
Source: BMC Psychiatry. 2023 Mar 2;23:132. doi: 10.1186/s12888-023-04619-w (PMC9983252; doi:10.1186/s12888-023-04619-w)
Supplement: Supplementary file 1 — Additional file 1. English version Questionnaire. [file 12888_2023_4619_MOESM1_ESM.docx]

**Additional files:**

**English version Questionnaire**

Questionnaire: English version on socio demographic, clinical, psychosocial, personal, and behavioral related factors of PLWHA on follow up.

**ST STUDY TITLE:** sleep quality and associated factors among HIV positive adults at follow up attending Dessie town governmental health facilities, Northeast Ethiopia, 2020.

Tabl

Part **1: socio-demographic related factors questionnaires**

**In instruction: Circle the number according the patients response**

| 1. Date: _____________   Code_____________ | | |
| --- | --- | --- |
| Q.NO | Questions | Response |
| 1. 101 | How old are you? | In_______years |
| 1. 102 | Sex? | 1. 1. Male 2. 2. Female |
| 103 | What is your marital status? | 1.Married  2.unmarried  3.Divorced  4.Widowed |
| 1. 104 | Residence? | 1. 1. Urban 2. 2.Rural |
| 1. 105 | What is your religion? | 1. 1.Orthodox 2. 2.Muslim 3. 3.Protestant4.Others(specify) -------------- |
| 1. 106 | What is your educational status? | 1. 1.unable to read and write 2. 2.Primary school 3. 3.Secondary school 4. 4.Diploma and above |
| 107 | What is your occupation? | 1. 1.Student 2. 2.Daily labour 3. 3.Farmer 4. 4.House wife 5. 5.Merchant 6. 6.Other (specify)_______ |
| 1. 108 | How much is your family monthly income (Ethiopian birr)? | ________________In Ethiopian birr? |

**Part II: Personal and behaviour related factors questionnaires circle the number according to patient’s response.**

| 201 | 1. Do you drink alcohol? | 1.Yes  2.No |
| --- | --- | --- |
| 202 | Do you smoke Cigarette? | 1. Never smoked  2. Current smoker  3. Past smoker |
| 203 | Do you chew Khat? | 1. Current chewer  2. Past chewer  3. Never chewer |
| 204 | Do you drink tea and coffee near to bed time? | 1.Yes  2.No |

**Part III: A clinical related factors questionnaires instruction fills in the blank space and choice the number and circles it according clients response.**

| 301 | What are the recent viral loads? | _____________Copies/ml | |
| --- | --- | --- | --- |
| 302 | What are The recent CD4 Cell counts? | ______________Cells/mm^3^ | |
| 303 | What is Current ART regimen? | _______________ | |
| 304 | When you Confirm being HIV positive? | In_____________year | |
| 305 | 1. Your Nutritional status? | | 1.weight _________In kg   1. 2.Height __________In meter |
| 306 | 1. Recent history of opportunistic infection? | | 1.Yes  2.no  3. If yes (specify) ___________? |
| 307 | 1. What is recent WHOHIV stage? | | ___________________? |
| 308 | 1. Is there currently present of any comorbidty disease? | | 1. Yes 2. 2.No 3. If yes (specify) _____________? |
| 309 | 1. What was your Current ART drug adherence? | | 1.Good  2.Fair  3.Poor |

**Part IV: Pittsburgh sleeping quality of index (PSQI) related questionnaires during the past one month.**

**Instructions:** The following **questionnaires** relate to your usual sleep habits during the past month only. Your answers should indicate the most accurate reply for the majority of days and nights in the past month. **Please answer all questions.**

- **From number 401 to 404.2 fill respondents answer in given space**

401**.** What time have you usually gone to bed at night? _____________in hrs

402. How long has it taken you to fall asleep each night? __________in minutes

403. What time you usually gotten up in the morning? ________ in hrs.

404 .1.How many hours of actual sleep did you get at night? __________in hrs

404.2. How many hours of actual sleep? ______________in hrs.

**For the table below Please insert the numbers according the clients respond in the given space** from question 405.1 to 407, (0=Not during the past month, 1=Less than once a week,2=Once or twice a week and 3=Three or more times a week and for question no408 and question no409 fill the no according to the given space.

| Q.no | Questions | Response | | | | | |
| --- | --- | --- | --- | --- | --- | --- | --- |
| 405 | During the past month how often have you had trouble sleeping because you | Not during the past month | | Less than once a week | Once or twice a week | | Three or more times a week |
| 1. 405.1 | Cannot get to sleep within 30 minutes |  |  | |  |  | |
| 405.2 | Wake up in the middle of the night or early morning |  |  | |  |  | |
| 405.3 | Have to get up to use the bathroom |  |  | |  |  | |
| 405.4 | Cannot breathe comfortably |  |  | |  |  | |
| 405.5 | Cough or snore loudly |  |  | |  |  | |
| 405.6 | Feel too cold |  |  | |  |  | |
| 405.7 | Feel too hot |  |  | |  |  | |
| 405.8 | Have bad dreams |  |  | |  |  | |
| 405.9 | I Have pain |  |  | |  |  | |
| 405.10 | Other reason(s),please describe___ |  |  | |  |  | |
| 406 | During the past month, how often have you taken medicine to help you sleep (prescribed or “Over the counter”)? |  |  | |  |  | |
| 407 | During the past month, how often have you had trouble staying awake while driving, eating meals or engaging in social activity? |  |  | |  |  | |
| 308 | During the past month, how much of a problem has it been for you to keep up enough enthusiasm to get things done | No problem at all  (0) | Only a slight problem  (1) | | Same what of problem  (2) | Avery big problem  (3) | |
|  |  |  |  | |  |  | |
| 309 | During the past month, how would you rate Your sleep quality overall? | Very  Good  (0) | Fairy  Good  (1) | | Fairy  Bad  (2) | Very  Bad  (3) | |

Part V: Perceive stress related questionnaires insert number according to patients responses (0=never, 1=almost never, 2=sometimes, 3=fairly often 4=very often).

| Q.no | Questionnaire | | Responses | | | | | |
| --- | --- | --- | --- | --- | --- | --- | --- | --- |
|  |  |  | Never | | Almost never | Sometimes | Fairly often | very often |
| 501 | How often have you been upset because of something that Happened unexpectedly? | |  | |  |  |  |  |
| 502 | How often have you felt that you were unable to control the important things in your life? | |  | |  |  |  |  |
| 503 | How often have you felt nervous and stressed? | |  | |  |  |  |  |
| 504 | | How often have you found that you could not cope with all the things that you had to do? |  | |  |  |  |  |
| 505 | | How often have you been angered because of things that Happened that been outside of your control? | |  |  |  |  |  |
| 506 | | How often have you felt difficulties were piling up so high that you could not overcome them? | |  |  |  |  |  |

**For table below insert numbers according to patients response :(4=never, 3=almost never, 2=sometimes, 1=fairly often 2= very often).**

| 507 | How often have you felt confident about your ability to handle your personal problems? | |  |  |  |  |
| --- | --- | --- | --- | --- | --- | --- |
| 508 | | How often have you felt that things were going your way? |  |  |  |  |
| 509 | | How often have you been able to control irritations in  Your life? |  |  |  |  |
| 510 | | How often have you felt that you were on top of things? |  |  |  |  |

**Part VI: Anxiety related questionnaires (0= Not at all 1=occasionally 2=a lot of and 3= Most of the time insert the number in the given space according to patients response but for question no604 circle the number).**

| Q.no | Question | Not at all | Occasionally | | A lot of time | | Most of the  Time | |
| --- | --- | --- | --- | --- | --- | --- | --- | --- |
| 601 | Do you feel tense or wound up? |  |  | |  | |  | |
| 602 | Do you get a sort of frightened feeling As if something Awful is about To happen? |  |  | |  | |  | |
| 603 | Do have Worrying thoughts Go through your  Mind? |  |  | |  | |  | |
| 604 | Can you sit  At ease and  Feel relaxed? | Definitely | Usually | | Not often | | Not at all | |
|  |  | 0 | 1 | | 2 | | 3 | |
| 605 | Do you feel a sort  Of frightened  Feeling like  ”butterflies” in  The stomach? |  |  |  | |  | |  |
| 606 | Do you feel  Restless as you have To be on The move? |  |  |  | |  | |  |
| 607 | Do you have a sudden  Feelings of  Panic? |  |  |  | |  | |  |

**Part VII: Depression related questionnaires for the giving table circle the number according to patient’s response.**

| 701 | Can things enjoy for you? | | Definitely as  much | | | Not quite  As much | Only a little | Hardly at all |
| --- | --- | --- | --- | --- | --- | --- | --- | --- |
|  |  |  | 0 | | | 1 | 2 | 3 |
| 702 | Can you laugh and See the funny side Of thing? | | | | As much As I always could | Not quite so  Much now | Definitely not  So much now | Not at  All |
|  |  |  |  |  | 0 | 1 | 2 | 3 |
| 703 | Do you feel  As cheerful? | | | | Most of  The time | Sometimes | Not often | Not at all |
|  |  |  |  |  | 0 | 1 | 2 | 3 |
| 704 | Do you feel as  you slowed  Down? | | | | Not at all | Sometimes | Very often | Nearly all  The time |
|  |  |  |  |  | 0 | 1 | 2 | 3 |
| 705 | | Do you have  Lost interest  Inyour appearance? | I take  Just as  Much care | | | I may not  Take quite  As much care | I don’t  Take as  Much care  As should | Definitely |
|  |  |  | 0 | | | 1 | 2 | 3 |
| 706 | | Can you look  Forward with  Enjoyment to  Things? | | As much  As I ever did | | Rather less  Than I used to | Definitely less than  I used to | Hardly at all |
|  |  |  |  | 0 | | 1 | 2 | 3 |
| 707 | | Can you enjoy  A good book  Or radio/program? | | Often | | Sometimes | Not often | Very seldom |
|  |  |  |  | 0 | | 1 | 2 | 3 |

| 801 | 1. Do you have sleep in noise environment? | 1. 1.Yes 2. 2.No |
| --- | --- | --- |
| 802 | 1. Do you live with your family | 1.Yes  2.No |
| 803 | Do Have separate room? | 1.Yes  2.No |
| 804 | Are you disclosed your status to your family? | 1.Yes  2.No |
